# Supplementary material for: Resource availability and barriers to delivering quality care for newborns in hospitals in the southern region of Malawi: A multisite observational study
Source: PLOS Glob Public Health. 2022 Dec 5;2(12):e0001333. doi: 10.1371/journal.pgph.0001333 (PMC10021306; doi:10.1371/journal.pgph.0001333)
Supplement: S1 Appendix — (DOCX) [file pgph.0001333.s002.docx]

**S1 Appendix: Health Facility Resource Survey tool**
