# Supplementary material for: Resource availability and barriers to delivering quality care for newborns in hospitals in the southern region of Malawi: A multisite observational study
Source: PLOS Glob Public Health. 2022 Dec 5;2(12):e0001333. doi: 10.1371/journal.pgph.0001333 (PMC10021306; doi:10.1371/journal.pgph.0001333)
Supplement: S1 Table — (DOCX) [file pgph.0001333.s003.docx]

**S1 Table:** **Standards of care and quality statements**

| **No.** | **Scope** | **Number of quality statements** | | |  |  |
| --- | --- | --- | --- | --- | --- | --- |
|  |  | **WHO MNH QoC Standards** | **Malawi MNH QoC Standards (adapted)** | **WHO Small and sick newborn (SSN) QoC standards** | **Quality measures assessed in this study for both MNH and SSN standards (inputs)** | **Findings/gaps identified through this study** |
| 1 | Evidence base practice | 9 | 10 | 42 | Up-to-date Clinical protocols for assessing women in all maternity unit ANC, pregnancy, labour and postnatal complications, infection control and management, essential newborn care, small and sick neonate, | Adequate clinical protocols for managing labour complications pasted on the walls of the labour and postnatal wards  Inadequate clinical protocols for neonatal resuscitation, infection prevention, care of small and preterm babies, care of the sick neonate and essential newborn care |
|  |  |  |  |  | In-services training of staff (once every year) in ANC, Pregnancy, labour and postnatal complications, essential newborn care, infection control and management, small and sick neonate, | Inadequate in-service training for staff |
|  |  |  |  |  | Prophylactic drugs in pregnancy, for babies (Vitamin K) in sufficient quantity all times | Vitamin K insufficient |
|  |  |  |  |  | Supportive supervision on management of cases | Inadequate supportive supervision by the facility leaders |
|  |  |  |  |  | Sterile cord ties and blades available all times | Inadequate sterile cord ties and blades in some hospitals |
|  |  |  |  |  | Essential resuscitation equipment and medication available all times | Always available in labour ward and neonatal unit |
|  |  |  |  |  | Oral and intravenous antihypertensive and magnesium sulphate all times | Available all times in 5 facilities |
|  |  |  |  |  | Functional laboratory for FBC, proteinuria, liver function test, urea, creatinine and electrolytes | Laboratory support for FBC |
|  |  |  |  |  | Essential supplies such as oxytocin in labour ward, IV fluids and blood administration supplies, baby breastfeeding and alternative feeding such as NGT | Oxytocin, intravenous cannula always available, but Intravenous fluids, giving sets, and nasal gastric tubes for babies not always available for use |
|  |  |  |  |  | Antibiotics for treating infection at all times | Inadequate antibiotics |
|  |  |  |  |  | Mechanisms for minimizing overcrowding, including one newborn per resuscitation unit, incubator or cot | Inadequate baby cots |
| 2 | Actionable information system | 2 | 2 | 3 | Facilities have registers, data collection forms, clinical and observation charts in place  at all times for routine recording and monitoring of all care processes for women and newborns | Registers available for newborn, KMC, antenatal, labour ward, postnatal, CCP and partograph |
|  |  |  |  |  | Conducted reviews of maternal, perinatal deaths at least once a month and mechanism in place to implement its recommendations | Only 2 hospitals conducted neonatal audits in previous month |
| 3 | Functional referral system | 3 | 3 | 6 | Ready access functioning ambulance or vehicle for referrals at all times | Inadequate ambulances for 6 hospitals |
|  |  |  |  |  | Reliable communication methods, including a mobile phone, land line or radio, which is always functioning | Mobile phone or land line always available for communication |
| 4 | Effective communication and family participation | 2 | 2 | 6 | Not assessed | Not applicable |
| 5 | Respective women and newborn care | 3 | 3 | 6 | Not assessed | Not applicable |
| 6 | Emotional, Psychological and Developmental support | 2 | 2 | 5 | Not assessed | Not applicable |
| 7 | Competent, motivated, empathetic multidisciplinary human resources | 4 | 3 | 4 | Skilled birth attendants and staff with skill mix available all times in sufficient number to meet anticipated workload. | Only nurses were available 24/7 |
|  |  |  |  |  | Regular training and mentorship | Inadequate training |
|  |  |  |  |  | Conducts annual appraisal for all staff | Annual appraisal not conducted |
|  |  |  |  |  | Facility has designated quality improvement team | Inactive quality improvement teams |
|  |  |  |  |  | Monitor quality improvement performance and communicate performance to facility staff | Neonatal outcomes pasted on the walls of only 4 hospitals |
| 8 | Essential physical resources for maternal, newborn /small and sick newborns | 6 | 3 | 6 | Facility has functioning source of safe water | Use of reservoir if no tap water |
|  |  |  |  |  | Facility has energy infrastructure (solar, generator, grid) at all times | Inadequate energy infrastructure in 6 facilities |
|  |  |  |  |  | Availability of equipped resuscitation equipment and supplies | Resuscitaire and bag and mask always available in labour ward and neonatal unit |
|  |  |  |  |  | Facility has neonatal ward/space for admitting sick neonates | All hospitals had wards/space for admitting sick neonates |
|  |  |  |  |  | Availability of essential antihypertensive agents, magnesium sulphate, uterotonic drugs, antenatal corticosteroids, first- and second-line antibiotics and supplies for intravenous infusion in sufficient amount at all times. | Magnesium sulphate was always available in 5 hospitals labour ward or postnatal ward  Dexamethasone not always available in labour ward  Both first- and second-line antibiotics were not always available |
|  |  |  |  |  | Equipment and supplies for detecting complications such as thermometers, sphygmomanometers, foetal scope, urine dipsticks, pulse oximeter in sufficient quantities all times | Thermometers were inadequate  Sphygmomanometers batteries not always available  Glucometer test stripes not always available |
|  |  |  |  |  | Essential laboratory supplies and tests (blood glucose, haemoglobin or packed cell volume, blood group and cross-matching, bilirubin, urine protein, full blood count, blood culture, electrolytes, renal and liver function tests, syphilis, HIV and malaria rapid  diagnostic tests | Facility laboratory not able to support bilirubin, PCV, Arterial blood gases and blood culture |
|  |  |  |  |  | A safe, uninterrupted oxygen source and delivery supplies (nasal prongs, catheters and masks), including nasal continuous positive airway pressure, available at all times in lab our, childbirth and neonatal areas (SSN) | Interrupted oxygen source due to inadequate electricity supply in 6 hospitals |
|  |  |  |  |  | Supplies and functioning equipment for emergency care and resuscitation of newborns (resuscitation table, well-stocked neonatal resuscitation trolley, warmer, suction device, pulse oximeter, laryngoscope) available all times | Resuscitaire and bag and mask, oxygen concentrator, CPAP and phototherapy always available in labour ward and neonatal unit but its functionality interrupted with inadequate electricity |
| 9 | Community health care and social accountability | N/A | 3 | N/A | Not assessed | Not assessed |
|  | **Total** | **31** | **31** | **78** |  |  |
