# Supplementary material for: Resource availability and barriers to delivering quality care for newborns in hospitals in the southern region of Malawi: A multisite observational study
Source: PLOS Glob Public Health. 2022 Dec 5;2(12):e0001333. doi: 10.1371/journal.pgph.0001333 (PMC10021306; doi:10.1371/journal.pgph.0001333)
Supplement: S2 Table — (DOCX) [file pgph.0001333.s004.docx]

**S2 Table: Total ambulances by facility**

| **Hospital** | **Available ambulance** | **Functional ambulance** | **% of functional ambulance** | **District^[[1]](#footnote-1)^ Population** | **Functional ambulance to population ratio** |
| --- | --- | --- | --- | --- | --- |
| ^[[2]](#footnote-2)^Hospital 1 | 6 | 3 | 50.0% | 851,737 | - |
| Hospital 2 | 9 | 5 | 55.6% | 721, 456 | 1:144,291 |
| Hospital 3 | 8 | 6 | 75.0% | 356,875 | 1:59,479 |
| Hospital 4 | 8 | 5 | 62.5% | 564,684 | 1:112,937 |
| Hospital 5 | 18 | 13 | 72.2% | 735,438 | 1:56,572 |
| Hospital 6 | 6 | 4 | 66.7% | 130,949 | 1:32,737 |
| Hospital 7 | 7 | 6 | 85.7% | 684, 107 | 1:114,017 |
| **Total** | **62** | **42** | **67.7%** | **4,045,246** | **1:81,885^[[3]](#footnote-3)^** |

1. Source: 2018 Malawi Population and Housing Census Report [↑](#footnote-ref-1)
2. Ration was not calculated as it’s a tertiary level facility and its population covers both city and rural. The district has district health office that manages transportation of referrals to tertiary hospital (hospital 1) [↑](#footnote-ref-2)
3. The total ambulance to population ratio excludes hospital 1 [↑](#footnote-ref-3)
