# Supplementary material for: Resource availability and barriers to delivering quality care for newborns in hospitals in the southern region of Malawi: A multisite observational study
Source: PLOS Glob Public Health. 2022 Dec 5;2(12):e0001333. doi: 10.1371/journal.pgph.0001333 (PMC10021306; doi:10.1371/journal.pgph.0001333)
Supplement: S3 Table — (DOCX) [file pgph.0001333.s005.docx]

**S3 Table: Training of Clinicians and Nurses^[[1]](#footnote-1)^**

| **Staff Training** | **Hospital 1** | | | **Hospital 2** | | | **Hospital 3** | | | **Hospital 4** | | | **Hospital 5** | | | **Hospital 6** | | | **Hospital 7** | | | **Total** |
| --- | --- | --- | --- | --- | --- | --- | --- | --- | --- | --- | --- | --- | --- | --- | --- | --- | --- | --- | --- | --- | --- | --- |
|  | LW | PNW | NW | LW | PNW | NW | LW | PNW | NW | LW | PNW | NW | LW | PNW | NW | LW | PNW | NW | LW | PNW | NW |  |
| **Total clinicians and nurses** | **21** | **16** | **16** | **8** | **7** | **7** | **17** | **6** | **6** | **15** | **8** | **6** | **15** | **13** | **10** | **11** | **0** | **4** | **20** | **13** | **6** | **225** |
| Integrated Maternal and Neonatal care (IMNC) | 20 | 9 | 0 | 0 | 4 | 0 | 14 | 1 | 3 | 0 | 0 | 0 | 0 | 0 | 0 | 0 | NA | 0 | 0 | 0 | 0 | 51 |
| **Proportion trained in IMNC** | **54.7%** | | | **18.2%** | | | **62.1%** | | | **0.0%** | | | **0.0%** | | | **0.0%** | | | **0.0%** | | | **22.7%** |
| Helping Babies Breath (HBB) | 20 | 0 | 1 | 0 | 4 | 0 | 14 | 1 | 1 | 0 | 0 | 0 | 11 | 0 | 0 | 0 | NA | 0 | 0 | 0 | 0 | 52 |
| **Proportion Trained in HBB** | **39.6%** | | | **18.2%** | | | **55.2%** | | | **0.0%** | | | **28.9%** | | | **0.0%** | | | **0.0%** | | | **23.1%** |
| Care of the Infant and Newborn (COIN) | 4 | 3 | 10 | 2 | 3 | 1 | 0 | 2 | 5 | 0 | 0 | 0 | 0 | 0 | 1 | 0 | NA | 0 | 0 | 0 | 4 | 35 |
| **Proportion trained in COIN** | **32.1%** | | | **27.3%** | | | **24.1%** | | | **0.0%** | | | **2.6%** | | | **0.0%** | | | **10.3%** | | | **15.6%** |
| Maternal and neonatal death audit | 20 | 3 | 10 | 0 | 3 | 5 | 0 | 1 | 6 | 0 | 0 | 2 | 0 | 0 | 0 | 0 | NA | 1 | 0 | 0 | 6 | 57 |
| **Proportion trained in death audit** | **62.3%** | | | **36.4%** | | | **24.1%** | | | **6.9%** | | | **0.0%** | | | **6.7%** | | | **15.4%** | | | **25.3%** |

1. Note: WHO quality of care standards for maternal and newborn care recommends all staff trained/refreshed in essential training once every 12 months [↑](#footnote-ref-1)
