# Supplementary material for: Resource availability and barriers to delivering quality care for newborns in hospitals in the southern region of Malawi: A multisite observational study
Source: PLOS Glob Public Health. 2022 Dec 5;2(12):e0001333. doi: 10.1371/journal.pgph.0001333 (PMC10021306; doi:10.1371/journal.pgph.0001333)
Supplement: S4 Table — (DOCX) [file pgph.0001333.s006.docx]

**S4 Table:** **Number of stockout days in the preceding month of essential supplies and drugs in pharmacy**

|  | **Hospital 1** | **Hospital 2** | **Hospital 3** | | **Hospital 4** | **Hospital 5** | **Hospital 6** | **Hospital 7** |
| --- | --- | --- | --- | --- | --- | --- | --- | --- |
| **Month** | **Jan-20** | **Jan-20** | **Jan-20** | **Feb-20** | | **Feb-20** | **Feb-20** | **Jan-20** |
| **Essential supplies** | | | | | | | | |
| Intravenous cannula size 24 | 0 | 0 | 0 | 0 | | 0 | 0 | 0 |
| Given sets (60 drop factor) | 0 | 0 | 0 | 0 | | 0 | 0 | 0 |
| Surgical blade for cutting cord | 0 | 0 | 0 | 0 | | 0 | 0 | 0 |
| Cord clamp | 23 | 31 | 0 | 14 | | 17 | 11 | 26 |
| Nasogastric tube size FG6/8 | 0 | 31 | 0 | 0 | | 0 | 20 | 0 |
| Nasal prongs | 0 | 31 | 0 | 23 | | 0 | 0 | 30 |
| Thermometers | 0 | 31 | 0 | 0 | | 0 | 0 | 0 |
| Blood Pressure calf | 0 | 31 | 0 | 0 | | 0 | 0 | 0 |
| **Essential drugs** | | | | | | | | |
| 50% dextrose | 0 | 24 | 0 | 28 | | 0 | 0 | 0 |
| Diazepam Intravenous | 0 | 0 | 0 | 25 | | 0 | 0 | 0 |
| Phenobarbitone Intravenous | 0 | 0 | 0 | 0 | | 0 | 0 | 0 |
| Magnesium Sulphate Intravenous | 0 | 0 | 7 | 10 | | 0 | 0 | 23 |
| Benzylpenicillin | 0 | 0 | 0 | 28 | | 1 | 10 | 0 |
| Gentamycin | 0 | 12 | 0 | 0 | | 0 | 0 | 0 |
| Ceftriaxone | 0 | 2 | 0 | 0 | | 0 | 0 | 30 |
| Oxytocin | 0 | 0 | 0 | 5 | | 0 | 0 | 0 |
| Dexamethasone Intravenous | 0 | 0 | 28 | 0 | | 0 | 0 | 0 |
| Vitamin K Intravenous | 0 | 30 | 0 | 0 | | 0 | 0 | 0 |
| Metronidazole Intravenous | 0 | 0 | 1 | 0 | | 0 | 0 | 0 |
| Artesunate Intravenous | 0 | 0 | 0 | 0 | | 0 | 0 | 0 |
| Aminophylline oral | 0 | 0 | 0 | 0 | | 10 | 24 | 0 |
