# Supplementary material for: Resource availability and barriers to delivering quality care for newborns in hospitals in the southern region of Malawi: A multisite observational study
Source: PLOS Glob Public Health. 2022 Dec 5;2(12):e0001333. doi: 10.1371/journal.pgph.0001333 (PMC10021306; doi:10.1371/journal.pgph.0001333)
Supplement: Attachment — Submitted filename: Response to Reviewers .docx [file pgph.0001333.s007.docx]

**PGPH-D-22-00596**

**RE: Response to Reviewers**

Dear Editor,

Thank you for giving us an opportunity to submit a revised draft of the manuscript “Resource availability and barriers to delivering quality care for newborns in health facilities in the southern region of Malawi: a multisite observational study” for publication in the PLOS Global Public Health Journal. We appreciate the time and effort that you and the reviewers dedicated to providing feedback on our manuscript and are grateful for the insightful comments on and valuable improvements to our paper. We have incorporated the suggestions made by the reviewers. The changes are highlighted within the manuscript. The revisions have been approved by all four authors. Please see below, a point-by-point response to the reviewers’ comments and concerns. All page numbers refer to the revised manuscript file with tracked changes.

Journal Requirements:

1. Please include a complete copy of PLOS’ questionnaire on inclusivity in global research in your revised manuscript. Our policy for research in this area aims to improve transparency in the reporting of research performed outside of researchers’ own country or community. The policy applies to researchers who have travelled to a different country to conduct research, research with Indigenous populations or their lands, and research on cultural artefacts. The questionnaire can also be requested at the journal’s discretion for any other submissions, even if these conditions are not met.  Please find more information on the policy and a link to download a blank copy of the questionnaire here: <https://journals.plos.org/globalpublichealth/s/best-practices-in-research-reporting>. Please upload a completed version of your questionnaire as Supporting Information when you resubmit your manuscript.

**Author Response:** Thank you very much for this guidance. We confirm that we have included inclusivity in global research questionnaire as a supporting document with submission of revisions. We have also included a statement in method section of revised manuscript page 9, lines 179-181 referring to this supplementary information (S1 Checklist).

2. Please amend your detailed Financial Disclosure statement. This is published with the article. It must therefore be completed in full sentences and contain the exact wording you wish to be published.

a. State the initials, alongside each funding source, of each author to receive each grant.
b. State what role the funders took in the study. If the funders had no role in your study, please state: “The funders had no role in study design, data collection and analysis, decision to publish, or preparation of the manuscript.”

**Author Response:** Thank you very much for this guidance. We have amended financial disclosure statement in the Revised manuscript on page 39, lines 716-720.

Additional Editor Comments (if provided):


[Note: HTML markup is below. Please do not edit.]

Reviewers' comments:

Reviewer's Responses to Questions

**Comments to the Author**

1. Does this manuscript meet PLOS Global Public Health’s [publication criteria](https://journals.plos.org/globalpublichealth/s/criteria-for-publication)? Is the manuscript technically sound, and do the data support the conclusions? The manuscript must describe methodologically and ethically rigorous research with conclusions that are appropriately drawn based on the data presented.

Reviewer #1: Yes

Reviewer #2: Partly

**Author Response:** Thank you for the feedback.

2. Has the statistical analysis been performed appropriately and rigorously?

Reviewer #1: Yes

Reviewer #2: Yes

**Author Response:** Thank you for the feedback.

3. Have the authors made all data underlying the findings in their manuscript fully available (please refer to the Data Availability Statement at the start of the manuscript PDF file)?

The [PLOS Data policy](https://journals.plos.org/globalpublichealth/s/data-availability) requires authors to make all data underlying the findings described in their manuscript fully available without restriction, with rare exception. The data should be provided as part of the manuscript or its supporting information, or deposited to a public repository. For example, in addition to summary statistics, the data points behind means, medians and variance measures should be available. If there are restrictions on publicly sharing data—e.g. participant privacy or use of data from a third party—those must be specified.

Reviewer #1: Yes

Reviewer #2: Yes

**Author Response:** Thank you for the feedback.

4. Is the manuscript presented in an intelligible fashion and written in standard English?

PLOS Global Public Health does not copyedit accepted manuscripts, so the language in submitted articles must be clear, correct, and unambiguous. Any typographical or grammatical errors should be corrected at revision, so please note any specific errors here.

Reviewer #1: Yes

Reviewer #2: Yes

**Author Response:** Thank you for the feedback.

5. Review Comments to the Author

Please use the space provided to explain your answers to the questions above. You may also include additional comments for the author, including concerns about dual publication, research ethics, or publication ethics. (Please upload your review as an attachment if it exceeds 20,000 characters)

Reviewer #1: Thank you for the opportunity to review this manuscript. It is very well written and organized. The topic area is important and practical. It is also very timely with the recent publication and dissemination of the new standards for sick and small newborn care as well as investment and interest in the health and survival of these vulnerable infants. This paper captures important information on the transition from policy to practice. I have a number of suggestions below that I think could strengthen this paper and highlight more concretely its added value. Thank you for conducting and writing up this work.

**Author Response:** Thank you for the feedback and suggestions to strengthen this paper

INTRODUCTION
- It would be helpful to provide a bit more context on what other related work has been done in Malawi and what gap this study fills / what is the added value. A couple studies worth looking at and discussing if you have not already: Kwaza 2020 BMC Health Services Research, Leslie 2016 PLoS, etc.

**Author Response:** Thank you for pointing this out and for the suggestion. We have included the section in the revised manuscript that explains what studies were done, gaps identified and what this study is adding. The paragraph is in the introduction section, pages 5-6, lines 91-98

RESULTS
- Summary: In the summary you note that an ambulance is available as a positive but throughout this is noted as a barrier. I suggest that you move this to the second paragraph of the discussion as refer to it as a barrier.

**Author Response:** Thank you for pointing this out and for the suggestion. We have removed a sentence from the summary of findings which showed ambulance availability as a positive. Instead, we have maintained the sentence in the second paragraph about inadequate ambulances. The changes are shown in the Discussion section, under the summary of findings, page 23, lines 296.

- I think it is important to mention that you have not looked into all aspects of the standards and why. For example, you do not discuss health information systems, demand side pieces, respectful care, etc.

**Author Response:** Thank you for pointing this out and the suggestion. We acknowledge that our study did not assess parameters such as actionable information system, effective communication and family participation, respective women and newborn care and emotional, psychological and developmental support. This was due to financial and time constraints. We have added a sentence in the revised manuscript under discussion, strength and limitations section, page 33, lines 506-510 to explain this point and the reason.

DISCUSSION
- Infrastructure: You say that there are inconsistencies with the WHO standards. Can you please elaborate a bit more on what those differences are to help the reader connect the standards with your data without having to reference the standards themselves? It would be good to have a short section summarizing the key standards relevant to this work somewhere in the paper and/or referencing them in a figure/table together or separate from the information gathered as part of this study.

**Author Response:** Thank you for pointing this out and the suggestion. We have included a table in supplementary information (S1 Table) which summarises the key standards relevant to this study in relation to the data captured in this study. We have also referenced it in the revised manuscript under discussion section, page 24, lines 300-301.

- Transport and communication: You write “The hospitals were compliant with the adapted standards having either a mobile phone, 263 landline or radio that functions all times.” However, in the results, you noted that “All hospitals had at least one functional landline telephone or mobile 165 phone at all times except hospitals 1 and 6, where the mobile phone was not always 166 available for use.” Please make sure these are consistent.

**Author Response:** Thank you for pointing this out and the suggestion. We acknowledge that the sentences were not consistent. We have revised the sentence under results section on page 12, lines 210-212 to show that the facilities were compliant with the adapted standards as it requires either a mobile phone or land line or radio always available for communication.

- Transport and communication: The last sentence – in the WHO standards? Where?

**Author Response:** Thank you for pointing this out. This last sentence is not in the WHO standards, but it is just explaining how useful referral and communication tools are in supporting quality obstetric and newborn care. We have made it clear in the revised manuscript that it is from the literature review and expert opinion survey conducted with 39 international experts from LMICs. The changes are shown in discussion section, under transport and communication, page 26, lines 342-343.

- How are these facilities generalizable to others in different regions in Malawi?

**Author Response:** Thank you for pointing this out. We acknowledge that generalisation may be limited and have included a sentence in a limitation section, emphasising caution in generalisability. We also discuss how the findings from this study might be of the use in other regions and LMICs with the similar context. Changes are in Strengths and Limitations section, page 32, lines 495-499.

- Can you please explain some of the potential reasons behind the differences between health facilities?

**Author Response:** Thank you for pointing this out. We have elaborated some of reasons for variations in discussion section, under guidelines and standard implementation, pages 31-32, lines 474-484.

- What about financial aspects and what role they plan in all of this?

**Author Response:** Thank you for pointing this out. We have elaborated how financial aspects impact the availability of resources as shown in discussion section, under guidelines and standard implementation, pages 31-32 ,lines 481-484.

Reviewer #2: The authors have written a concise description of the readiness among a sample of hospitals in southern Malawi to provide essential newborn care. Overall, the manuscript is clearly written and the study is robust in having used a standard tool adapted to Malawi to measure readiness, one which apparently was used in other related QI work in Malawi. The team has used some strong data visualizations to synthesize a range of data, however there are areas where a reader is still left not quite sure of the results. The paper is important in further adding to the evidence of the need to translate policy into resources needed as a first step to ensuring quality and equitable care. As such, I had a number of suggestions which I believe would strengthen the manuscript and its value to the broader community working to improve outcomes for newborns and reduce still births

**Author Response:** Thank you for the feedback and suggestions to strengthen this paper


Introduction
The authors describe the work by WHO establishing standards and a process for improvement. For someone less fluent, it would be helpful to use a few sentences to detail the guidance and the 7 steps. This will also help interpret the results in the broader context. Similarly, any existing data on hospital (and health center) readiness from Malawi would frame this study, as well as patterns of care including FBD, post-natal care and any insights into timing and causes of neonatal deaths in Malawi (ex. In-hospital versus post-discharge) as well as any data from the audits described. They also describe an additional area of CHWs, but this is not assessed nor discussed.

**Author Response:** Thank you for pointing this out and suggestions. We have revised the introduction section to include few sentences that are explaining about the 8 standards in a lay summary as shown in the introduction section, page 6, lines 111-118. We have also included a table in supplementary information (S1 Table) which summarises the standards for maternal and newborn care as well as for small and sick babies.

We have also included a section that describe previous studies that assessed readiness and the gap which this study is trying to address, as shown on pages 5-6, lines 91-98.

More information has also been added on facility birth delivery (FBD if this means that) postnatal care gaps, timings and causes of neonatal deaths and challenges in the community and facility. The changes are shown from pages 4-5, lines 70-86. This study did not collect data from the community which we have included as a limitation as communty level factors, such as delay in seeking care are relevant to in-hospital deaths. Changes on under discussion chapter, page 33 line 510-512.

Methods:
It was interesting that the survey was done during COVID-19-what if any impact and /or precautions were taken?

**Author Response:** Thank you for pointing this out. We would like to clarify that this component of the facility survey was completed between January and February 2020 before COVID 19 was declared in Malawi (April 2020). But as indicated in the introduction section, page 7, lines 126-128, this study was conducted in the context of the larger study that ran from August 2019-December 2020. So, no COVID-19 precautions were in place during the time this survey component was conducted. We have made necessary changes in the abstract on page 2, lines 29-30 and methods section, page 8, line 156 to reflect the exact period this survey was conducted.

It was also a little unclear why these were chosen and why HCs (who likely encounter significant stillbirth and need to transfer sick young infants). This also needs to be in the limitations

**Author Response:** Thank you for pointing this out. We assume HCs means Health Centres, if so, we would like to explain that this study did not collect any data in the health centres. We collected data in 1 tertiary facility (central hospital) and 6 secondary level facilities (district hospitals). Health centres were mentioned when introducing what care central or regional hospitals and district hospitals offer in the methods section, page 7 lines 132-140. The district hospitals have been equipped to provide specialised neonatal care at district level with only few complicated cases referred to tertiary level facilities in this context. We have also mentioned in Methods section page 7 lines 139-140 why health centres were excluded. We chose district hopsitals and 1 central hospital that were performing stillbirth or neonatal death audit during study period.

Did the survey also capture C-section capacity as an important approach to reduce stillbirths? What were the components for managing sick- young infants (I saw CPAP, KMC support? Feeding?

**Author Response:** Thank you for pointing this out. We acknowledge that C-Section capacity was not captured in this survey, and we have included it as a limitation on page 32 lines 499-500.

For the components for managing sick-young infants, we captured availability of CPAP, KMC support, feeding, resuscitation, phototherapy and laboratory support as indicated in table 3 and 4. Pages 17-20.


Results
The results are details and a bit more information would be helpful for a reader. Many of the results are described qualitative, requiring looking at the table or appendix. Adding in some numbers/range to the text would be helpful (ex. Essential supplies). Similarly, when the term “sometimes” is used, need to define in methods what that means (anything between none and always?)-particularly for Table 2

**Author Response:** Thank you for pointing this out and the suggestion. We edited the essential and supplies section to include numbers and range. The changes are shown on page 15 under Essential supplies, drugs and equipment, lines 230-246.

We have added the definitions for “Always, Sometimes and Never” in the methods section page 8 lines 160-166 and in Table 2 footnote on page 13.

In table 1-some measure of volume would help understand the workload by hospital. It would also be helpful to have the number as well as % for positions filled (and was this overall versus larger or smaller gaps in some areas?
Why were only 3 district hospitals allocated ambulances? This should be discussed in the discussion. Also, I did not see anything about functioning of the referral system (both to and from the hospitals) beyond ambulance and phone availability. Were there metrics in the adapted survey which asked about things like counter referrals or feedback?
**Author Response:** We appreciate you bringing this up and making the proposal. The volume measurement has been added to Table 1. The number of deliveries and newborn admissions that the facilities have annually has been added. These data were gathered in a separate study that was part of the larger study on which this analysis is based. The updated manuscript's Table 1 on page 11 reflects the changes. We are aware that the data on positions filled was only presented using a percentage. We want to clarify that this information was taken from a Malawian Human Resource for Health document (2018); it was not collected by us. To ensure that we only have the data we gathered, we removed it from the table. The discussion section under the heading "Staff Availability and Training" on page 26, lines 354–361, discusses the information regarding the positions filled from source document.

Potential reasons for allocating ambulances to only 3 hospitals have been described in the Discussion section pages 31-32 lines 476-484 from the findings from another separate study under the main study which is unpublished.

We acknowledged that we did not assess further the referral feedback system both to and from the hospitals apart from transportation and communication. We acknowledge it as a limitation and we have added in the limitation section, page 33, lines 510-512.

Similarly-since hospital 6 combined their post-natal ward-I was confused why authors did not assess the combined ward for both competencies and staffing since PNC was being delivered
Did any of the hospitals have a specific area for sick and LBW infants (even if not officially a NICU?

**Author Response:** Thank you for pointing this out. We agree that postnatal care was offered at hospital 6 despite been combined. We have corrected throughout the manuscript as what we found in the labour ward of hospital 6 also applies to the postnatal ward. Changes are shown in results section, page 9, lines 185-186, Table 2, page 13 and Table 3, pages 17 - 19.

Responding to specific care for sick and LBW infants; yes, the hospitals had separate wards for sick and LBW infants, and this is what is referred to as nursery/neonatal wards throughout the manuscript.

Table 2 is a nice way to visualize data. Is that any versus none?

**Author Response:** Thank you for pointing this out. This table presents assessment of staff availability per cadre per shift in 24 hours as described on page 12, lines 214-217. It is not any versus none, but which cadre is available per shift (“Always” means that the cadre is available at that shift and represented by green colour. “Sometimes” means the cadre is available some of time but not the whole shift and this is represented by yellow colour. While “Never” means no such cadre is available during that shift and is represented by red colour. We have also provided a description of the meaning of “Always, Sometimes and Never” in the footnote of table 2, page 13 and methods section as requested in one of the comments above, page 8, lines 160-166.

The training gaps are well noted, but the standard is not discussed until in discussion. It would be helpful to have in an appendix (or noted in the text) what is the standard, so a reader knows in results where there are gaps. IN the discussion As the authors point out, there were gaps in required CPD (although also limited if any evidence on impact), What dd the MOH supervisors do?

**Author Response:** Thank you for pointing this out and for the suggestion. We have included standard requirements in the results section page 12 line 217-219, and a footnote is inserted in Supplementary information (Table S3) page 12.

On CPD gaps, we did not assess CPD functions in this study. In this section (discussion section), CPD is discussed, drawing from a previous study that was conducted in Kenya. It was a reference point in a discussion that might affect the training of staff in Malawi as well. We did not assess what the MoH Supervisors did as it was out of the scope of this study, but only assessed if supervisions were conducted.

For supplies, Why is oxytocin needed in neonatal ward? Was there no HCT but full blood count? Was bilirubin available through sending out?

**Author Response:** Thank you for pointing this out. On oxytocin, we did not assess its availability in neonatal wards which is why we put N/U in Table 3 and described with a note in the table that NU means the drug or supply was not used in postnatal or neonatal ward but only in Labour ward on page 17 and 18. The facilities could check full blood count but not PCV. We did not find out if bilirubin was available through sending out.

Were there any summary scores for the areas measured and specifically for care of sick young infants (some emerging approaches for this)

**Author Response:** Thank you for these questions. We assessed resource availability including equipment to support CPAP, resuscitation, ward warmth and feeding. These components are reported in the neonatal ward column in the tables in the results section. We are not aware of any validated summary scores of these data. We did not measure the process of care as it was not within the scope of this study.

Discussion
The authors have a nice description of the findings, but I would like to see a bit more digging into why and possible solutions from the growing work and literature to improve neonatal care in Malawi (ex. Makonde, NEST) and the region.

**Author Response:** Thank you for pointing this out and the suggestion. We have added in the discussions some of the reasons and possible solutions through initiatives that are already in place and link to studies done within the region. The changes are shown discussion section pages 24-30, lines 307-452.

Some specific comments:
P20 they note that clinicians were not always available during night shift-was this across all 3 areas? What was their workload?

**Author Response:** Thank you for pointing this out. We have added the text to indicate that clinicians were not always available during night shift and the work load they experienced when on call during the night. The changes are shown on page 26-27, lines 365-369.

See comment above about CPD. The sentence calling for more studies of CPD on outcomes seems odd given the extensive evidence that training and retraining alone is not effective but must include supportive supervision (see Rowe)

**Author Response:** Thank you for pointing this out and the suggestion. We have edited the sentence about CPD and now it reads: “As training alone is not effective in improving quality of care, we recommend inclusion of supportive supervision from both national and district level as suggested by Rowe and colleagues” Changes are reflected on page 28, lines 389-391.

On page 22, they have a bit if a value comparison with Ethiopia and a systematic review -It would be good to have a bit more granularity-what was similar and different at the hospital level? Any insights into variability between the surveyed hospitals (this does need more discussion)

**Author Response:** Thank you for pointing this out and the suggestion. We have added a discussion on referenced studies to show differences and similarities. The changes are shown on page 29, lines 420-427

The section on p22 on supervision is a nice background on why it is important, but no discussion on what the MOH does, and what the findings in Malawi compare with other published work
**Author Response:** Thank you for pointing this out. We have added a discussion on what MOH does and findings about supervision in Malawi and other published work The changes are shown on page 30 lines 438-447.

I was curious the reasoning behind a larger survey needed, versus one with plans and resources to address the gaps. I was also interested in who the community components could be measured (as I think it was not in the modified SARA)

**Author Response:** Thank you for pointing this out. We have removed the recommendation of larger survey as we acknowledge that is unnecessary as compared to resources surveys that will identify immediate needs to address. The changes are reflected on page 31, lines 467-469

I would add to the limitations that observation/delivery of care was not included (see Das et al for the gap between inputs and delivery) and no data on the community components nor on the QI. I think also missing is How to understand the readiness needed to prevent still birth and where that overlaps and differs for neonatal (in hospital and post discharge)

**Author Response:** Thank you for pointing this out and the suggestion. We have added to the study limitation section gaps in relation to the observation of delivery of care, missing of community components, QI and caesarean capacity assessment. The changes are reflected on pages 33, lines 510-512. We have also included a limitation that this study broadly discussed readiness available for stillbirths and neonatal deaths not necessary differentiating from stillbirths and neonatal deaths or showing overlap. The changes are shown in discussion section under strengths and weakness, page32, lines 500-503.
